# Blood pressure and noncommunicable diseases in middle-aged and older adults in China

**DOI:** 10.1371/journal.pone.0206635

**Published:** 2018-11-02

**Authors:** Yinghui You, Jincai Wang, Wenjie Teng, Guifeng Ma, Pengtao Liu

**Affiliations:** Weifang Medical University, Weifang, Shandong Province, China; Shanghai Institute of Hypertension, CHINA

## Abstract

**Background:**

There are few studies examining the association between blood pressure (BP) and noncommunicable diseases (NCDs) in consideration of the new hypertension guidelines in China.

**Methods:**

Data were drawn from the China Health and Retirement Longitudinal Study. 14 390 eligible participants (aged 45 years and older) were selected through four-stage, stratified, and cluster sampling. Hypertension was considered as a mean systolic blood pressure (SBP) of 130 mm Hg (old definition: 140 mm Hg) or higher, a mean diastolic blood pressure (DBP) of 80 mm Hg (old definition: 90 mm Hg) or higher or taking anti-hypertensive medication. Cochran-Armitage trend test and logistic regression analyses were conducted to test the association between BP level and NCDs.

**Results:**

The prevalence of hypertension based on the latest definition was 56.35% (while by old definition: 42.75%). The awareness, treatment, and control among hypertensive participants were 38.62% (51.18%), 43.10% (56.81%), and 9.91% (13.06%), respectively. An increasing rate of NCDs (diabetes, heart disease, stroke, and memory-related disease) among participants were found with the ascending of BP level. After adjusted for demographics and behavioral risks, the following 3 NCDs had been shown to correlate with hypertension: diabetes (adjusted OR 1.15, 0.91–1.45 for elevated BP; 1.20, 0.97–1.49 for hypertension stage 1; 1.55, 1.28–1.86 hypertension stage 2), heart disease (0.94, 0.79–1.12; 1.05, 0.90–1.22; 1.28, 1.12–1.47), and stroke (1.77, 1.25–2.51; 1.32, 0.93–1.87; 1.85, 1.37–2.49).

**Conclusions:**

The association between hypertension and the risk of NCDs is of concern in China. The combined efforts on NCDs prevention and lowered blood pressure should be made by nationally integrated strategies, especially in middle-aged and older adults.

## Introduction

The threat of noncommunicable diseases (NCDs) has been recognized globally and the World Health Organization (WHO) has set a target to reduce the overall mortality (from the 2013 baseline) from cardiovascular diseases, cancer, diabetes and chronic respiratory diseases by 25% by 2025 [1]. In the past few decades, NCDs have emerged rather than infectious diseases as the leading causes of death in China [2–4]. Among them, hypertension has been identified to be the second largest risk factor causing disability and death in China and the third leading risk factor of the total burden of diseases [5, 6]. This is convinced in the studies from China during the last thirty years which have reported an increasing prevalence of hypertension [7–9]. A cohort study from China showed that one third Chinese adults had hypertension and the prevalence increased with age (from 12.6% at 35–39 years of age to 58.4% at 70–74 years of age) [7]. Observational studies have demonstrated that unusual blood pressure (BP) is strongly and directly related to vascular (and overall) mortality throughout middle and old age [10]. The high BP-related increase is larger in older persons given the higher absolute risk of NCDs [11]. If left untreated or uncontrolled, hypertension will be responsible for many risks of NCDs at an older age [5, 12].

Although some studies have documented the high prevalence of hypertension in China [13–15], most of them were based on previous definition of hypertension, and the cutoffs for hypertension had been a top number of 140 mm Hg systolic and a lower number of 90 mm Hg diastolic. Recently, new medical guidelines defined hypertension as BP higher than 130 over 80 mm Hg, which was issued by the American Heart Association (AHA) in November 2017 [11]. We adopted it to make contrast with the old definition of hypertension and analyze the subsequent different results, but whether this definition is suitable for the prevention of NCDs in Chinese adults remains to be studied in the future.

China has more older people than other countries, and its ageing population is one of the largest in the world [16]. Too many older people will be affected by the new diagnostic criteria for hypertension. Therefore, more robust evidence is needed to demonstrate a causal link between the new diagnostic criteria for hypertension and NCDs in China. This paper compares the previous and the latest definition of hypertension and analyzes the association between BP level and NCDs in middle-aged and older adults in China.

## Methods

### Ethical approvals

Ethical approval for the study was granted by the Ethical Review Committee of Peking University, and all the participants provided written consent at the time of participation. All methods were performed in accordance with the relevant guidelines and regulations.

### Study design and target population

CHARLS is a nationally representative longitudinal survey among middle-aged and older adults in China [17]. It is designed to examine health and economic adjustments to rapid ageing population in China, using a four-staged, stratified and cluster sampling method. Details of the sampling methodology and the core CHARLS questionnaire have been described in previous studies [17, 18]. We used data from the national follow-up survey data of CHARLS (version ID: 20171011). Eligible individuals at the age of 45 and older were selected for this analysis with complete data on anthropometric measurements, health outcomes and individual weights. All the participants had blood pressure measurements taken.

### Definitions

Hypertension was defined in accordance with the 2017 High Blood Pressure Clinical Practice Guideline issued by the AHA in November 2017 [11]. Blood pressure was measured by a trained investigator (equipment: Omron HEM-7200 Monitor, Batteries, and Stopwatch). Participants were required to rest for at least 5 minutes in a seated position before the measurement. During the measurement, participants were required to insert the arm cuff plug into jack on the side of the monitor, place the cuff on left arm approximately 1.2 cm above the elbow, and position the arrows over the brachial artery on the inside of the arm. Three BP measurements (at 5-minute intervals) were obtained from each participant, according to the common protocol recommended by AHA [19]. Participants were deemed to have hypertension if the results of examination indicated that, based on the average of three measurements, the systolic blood pressure (SBP) was ≥130 mm Hg, or the diastolic blood pressure (DBP) was ≥80 mm Hg, or if they reported current use of antihypertensive medication. Participant with hypertension was considered “awareness” if he/she gave a positive response to the question, “Have you been diagnosed with hypertension by a doctor”. BP is categorized into four levels: Normal BP is defined as SBP <120/ DBP <80 mm Hg; elevated BP 120-129/<80 mm Hg; hypertension stage 1 is 130–139 or 80–89 mm Hg, and hypertension stage 2 is ≥140 or ≥90 mm Hg [11]. Treatment of hypertension was defined as the use of a prescription medication (Taking Chinese traditional medicine or taking Western modern medicine) for management of high BP at the time of interview. Control of hypertension was defined as pharmacological treatment of hypertension associated with an average SBP ≤130mm Hg and DBP ≤80 mmHg.

NCDs included self-reported data on whether the respondent had been diagnosed with diabetes or high blood sugar, cancer or malignant tumor (excluding minor skin cancers), chronic lung diseases, such as chronic bronchitis, emphysema (excluding tumors, or cancer), liver disease (except fatty liver, tumors, and cancer), heart disease (defined as heart attack, coronary heart disease, angina, congestive heart failure or other heart problems), stroke, kidney disease (except for tumor or cancer), stomach or other digestive disease (except for tumor or cancer), emotional, nervous or psychiatric problems, memory-related disease, arthritis or rheumatism, and asthma. The diagnosis was made by the trained doctors.

Body mass index (BMI) was calculated as weight in kg/height^2^ in m^2^, and classified as normal weight (BMI 18.5–23.9), overweight (BMI 24.0–27.9) and obesity (BMI ≥ 28.0) by Chinese standards [20]. Smoking status indicated whether the respondent reported ever or current smoking. Smoking included pipe smoking, tobacco chewing, self-rolled cigarettes, cigars and cigarettes. Drinking frequency was surveyed by asking the respondent about their use of alcohol (liquor, beer, or wine) and how often they drank the alcohol during the past year.

### Statistical analysis

Cochran-Armitage trend test was used to measure the association between the NCDs (considered as the response) and the BP categories. NCDs increased with BP in the Cochran-Armitage trend test were analyzed in the multivariable logistic regression adjusted for demographics, behavioral risks, and current residence. We used the biomarker weight, which was calculated based on the individual weights and the individual responses in biomarker [17, 18]. All the statistical analyses were performed with R 3.4.1 and a P value lower than 0.05 was considered significant.

## Results

### Descriptive findings

A total of 14 390 participants were included in this study. In terms of the old hypertension guideline (SBP ≥ 140 OR DBP ≥ 90) and the new hypertension guideline (SBP ≥ 130 OR DBP ≥ 80), the prevalence of hypertension and the awareness, treatment, and control among hypertensive participants were shown respectively in Table 1. The prevalence was higher among drinkers and participants with major NCDs listed in Table 1. The hypertension prevalence increased with age and BMI (Table 1).

**Table 1:** The weighted hypertension prevalence of the study participants (n = 14390).

Variables	%Hypertension (new guideline)	p	%Hypertension(old guideline)	p
Total	56.46		42.75	
Awareness (%)	38.74		51.18	
Taking medicine (%)	43.01		56.81	
Control (%)	9.89		13.06	
Age (%)		<0.0001		<0.0001
45–54	46.19		30.10	
55–64	55.95		42.51	
65–74	65.58		54.87	
≥75	74.02		62.50	
Gender (%)		<0.0001		0.3254
Male	58.38		43.30	
Female	54.56		42.20	
Residence (%)		0.0875		0.0113
Main city zone	60.07		46.90	
Others*	54.65		42.87	
Village	55.73		41.19	
BMI (kg/m^2^)		<0.0001		<0.0001
<18.5	41.46		30.68	
18.5–23.9	48.13		34.52	
24–27.9	62.43		48.16	
≥28	76.62		62.86	
Sleep duration		0.0932		0.0064
<7 h sleep	56.60		43.45	
7–10 h sleep	55.66		41.11	
≥10 h sleep	56.38		44.52	
Smoking (%)		0.0027		0.7312
Never	58.65		43.79	
Ever or current	54.95		42.03	
Drinking frequency		<0.0001		<0.0001
Never	51.44		36.90	
<1 drink/month	59.95		43.61	
≥1 drink/month	55.71		43.28	
Dyslipidemia		<0.0001		<0.0001
No	54.87		40.41	
Yes	69.86		62.42	
Diabetes or high blood sugar		<0.0001		<0.0001
No	55.43		41.24	
Yes	73.59		67.63	
Cancer or malignant tumor		0.5104		0.0376
No	56.42		42.64	
Yes	61.13		53.04	
Chronic lung diseases		0.0011		<0.0001
No	56.23		42.28	
Yes	58.59		47.01	
Liver disease		0.6610		0.0701
No	56.36		42.45	
Yes	58.74		49.04	
Heart disease		<0.0001		<0.0001
No	54.63		40.08	
Yes	70.80		63.55	
Stroke		<0.0001		<0.0001
No	55.79		41.91	
Yes	82.51		75.25	
Kidney disease		0.4820		0.0009
No	56.12		42.17	
Yes	61.48		51.13	
Stomach or other digestive disease		<0.0001		0.2037
No	57.69		43.30	
Yes	52.07		40.77	
Emotional, nervous, or psychiatric problems		0.4517		0.4932
No	56.55		42.76	
Yes	49.92		41.99	
Memory-related disease		<0.0001		<0.0001
No	56.23		42.47	
Yes	73.78		63.64	
Arthritis or rheumatism		0.0002		<0.0001
No	55.49		40.59	
Yes	58.60		47.47	
Asthma		0.0068		0.0022
No	56.31		42.52	
Yes	60.67		48.63	

Note:

*Other includes: combination zone between urban and rural areas, the town center, and special area, and township central; all numbers were weighted with the cross-sectional biomarker weights.

### Association between the prevalence of NCDs and blood pressure

**Fig 1:**
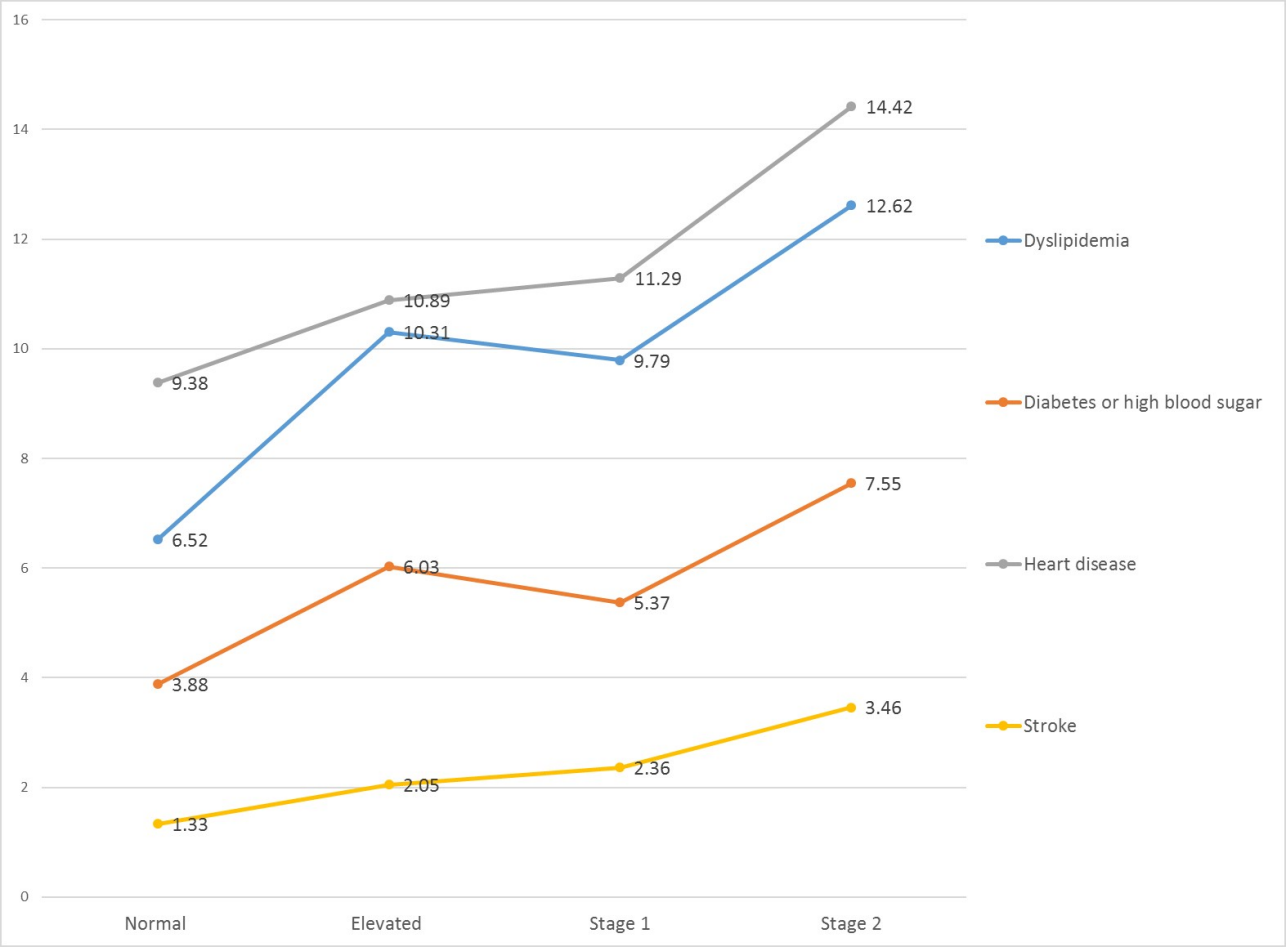
Associations between the prevalence of NCDs and increased blood pressure.

The prevalence of dyslipidemia, diabetes or high blood sugar, heart disease, and stroke increased with BP level (Fig 1). The increasing trend of other NCDs was not significant.

### Results of logistic regression models describing the association between NCDs and blood pressure levels

Table 2 shows the association (OR, AOR and relevant CI) between NCDs and BP. After adjusted for demographics, BMI, behavioral risks, and current residence, the following 3 chronic diseases to correlate with hypertension and elevation of BP had been shown in detail: diabetes, heart disease, and stroke. No evidence was found for an increased rate of cancer, lung disease, liver disease, digestive disease, emotional, nervous, or psychiatric problems, and asthma among hypertension participants.

**Table 2:** Results of logistic regression models describing the association between NCDs and blood pressure levels among participants.

NCDs	Diabetes				Heart disease				Stroke			
Variables	OR(95% CI)	P	AOR(95% CI)	P	OR(95% CI)	P	AOR(95% CI)	P	OR(95% CI)	P	AOR(95% CI)	P
Hypertension												
No	1.00		1.00		1.00		1.00		1.00		1.00	
Yes	**2.30(1.95,2.70)**	< .0001	**1.79(1.50,2.13)**	< .0001	**2.01(1.79,2.25)**	< .0001	**1.58(1.39,1.79)**	< .0001	**3.27(2.46,4.34)**	< .0001	**2.73(2.04,3.67)**	< .0001
Hypertension (old)												
No	1.00		1.00		1.00		1.00		1.00		1.00	
Yes	**3.03(2.60,3.54)**	< .0001	**2.13(1.80,2.53)**	< .0001	**2.59(2.32,2.89)**	< .0001	**1.90(1.68,2.14)**	< .0001	**3.98(3.07,5.14)**	< .0001	**3.18(2.42,4.17)**	< .0001
Blood pressure (BP)												
Normal BP	1.00		1.00		1.00		1.00		1.00		1.00	
Elevated BP	**1.27(1.01,1.59)**	0.9379	1.15(0.91,1.45)	0.5129	1.07(0.90,1.26)	0.1339	0.94(0.79,1.12)	0.0498	**2.04(1.46,2.84)**	0.0706	**1.77(1.25,2.51)**	0.0622
High BP/Stage 1	1.20(0.98,1.48)	0.4259	1.20(0.97,1.49)	0.9356	1.13(0.97,1.30)	0.5269	1.05(0.90,1.22)	0.8139	**1.69(1.23,2.33)**	0.9619	1.32(0.93,1.87)	0.4216
High BP/Stage 2	**1.72(1.44,2.06)**	< .0001	**1.55(1.28,1.86)**	< .0001	**1.52(1.33,1.73)**	< .0001	**1.28(1.12,1.47)**	< .0001	**2.34(1.76,3.10)**	< .0001	**1.85(1.37,2.49)**	0.0052

**Note**: The significant influencing factors of NCDs were in bold font. AOR: adjusted for age, gender, BMI, sleep duration, behavioral risks, smoking status, drinking frequency and current residence.

## Discussion

Recently, according to the new definition of hypertension issued by the AHA, the number of Chinese aged 45 years and older with high hypertension jumped from 42.75% to 56.46%. While with the old definition (the cutoffs for high blood pressure had been a top number of 140 and a lower number of 90), the awareness, treatment, and control among hypertensive participants were 51.18%, 56.81%, and 13.06%, respectively, according to the new definition (the cutoffs for high blood pressure had been a top number of 130 and a lower number of 80), the awareness, treatment, and control among hypertensive participants were 38.74%, 43.01%, and 9.89%, respectively. The results showed that the number of Chinese middle-aged and older population in hypertension was increasingly larger and the control of hypertension was surprisingly lower in terms of the new definition. We also calculated rates of hypertension stratified by baseline characteristics to allow for direct comparisons (Table 1). The new hypertension rate stratified by baseline characteristic was always higher than the rate according to the old definition, which suggested that more and more middle-aged and older Chinese people would be infected by the new hypertension guideline. If patients with hypertension are poorly controlled, they will face an increasing risk of developing many NCDs and are at an increasing risk of mortality from these diseases [7]. So early identification, treatment and prevention of hypertension should be considered to help increase the control rate of hypertension and then effectively reduce the higher prevalence of hypertension in the above population.

In this study, the middle-aged and older adults in China suffering high prevalence of hypertension with low awareness tend to carry a high burden of NCDs. This is consistent with some studies which demonstrate that hypertension is an independent risk factor for cardiovascular disease, myocardial infarction, and stroke [11, 21–24]. However, to our knowledge, most of these studies used the old definition of hypertension or focused on a few specific NCDs, and no national survey of most major NCDs had been done to test the implementation of the new definition of hypertension in a developing country. Therefore, in the present study, we used a standard design, which was harmonized with leading international research studies in the Health and Retirement Study (HRS) model, for a population survey and four-staged, stratified and cluster sampling method to ensure the representativeness of our study [17]. The results showed that the prevalence of NCDs including diabetes, high blood sugar, heart disease, stroke, and memory-related disease were markedly increasing with the ascending of BP, from levels as low as 120 mm Hg systolic and 80 mm Hg diastolic among individuals aged 45 and older in China (Fig 1). After adjusted for demographics and behavioral risks, the prevalence of NCDs including diabetes, heart disease, and stroke had been shown to closely correlate with hypertension.

In addition, the results of this study showed that the prevalence of diabetes and heart disease were significantly related to overweight. Participants who had higher BMI were more likely to suffer hypertension, diabetes and heart disease. This finding was largely similar to some studies which showed when compared with normotensive individuals, individuals with elevated BP were more likely to be overweight and obese, to progress to established hypertension, and to experience premature NCDs [25]. After adjusted for BMI, lower risk level of elevated BP was found in most of the NCDs. So, measures in reducing the prevalence of NCDs in China could be put forward after knowing risk factors of NCDs associated with BP. Lifestyle modification to have healthy weight and normal BP is one of the recommendations for all individuals with elevated BP to reduce the prevalence of many NCDs. Further studies are required to confirm the anticipated benefits of identifying and intervening in persons with elevated BP.

In this study, female participants had lower prevalence of hypertension than male. However, they were more likely to have heart disease. This is consistent with previous studies that gender differences had been described as an important factor for cardiovascular risk factors and cardiovascular risk status and the total number of deaths from cardiovascular diseases was greater among older women than among men [26]. One study found that physical activity tended to be lower among women than among men [27]. Another study found that women had a higher prevalence of obesity than men [28]. Taken together, these behavioral differences suggested that competing healthy behavioral factors (e.g., physical activity, obesity) might increase the disparity of the gender gap in heart disease. Further studies will be needed to confirm the effect of gender in related NCDs among Chinese older adults.

The results in our study also showed that the prevalence of NCDs was higher in main city zone than in other areas. This result may be due to different living and working conditions between main city zone and other areas in China. Urbanization led to social and economic progress, but also brought about a series of problems, such as heavy work pressure, irregular life style, serious environment pollution, traffic block and housing shortage, etc. This finding agreed with several previous national or regional studies which had documented that the prevalence of NCDs increased substantially with economic development in China [29, 30]. We suggest these middle-aged and elderly people who are targeted by new definition of BP raise their awareness of prevention of NCDs with regular and healthy life style. The Chinese government should spare no effort to improve the quality of environment and people’s basic living standards and help the older raise awareness of harmful high blood pressure. What’s more, other nationally integrated strategies are also needed to improve the prevention and control of hypertension and NCDs in China.

Our study has several limitations. First, it was limited by its cross-sectional nature. We just simply analysed the relationship between BP and NCDs, and no further analysis to find the original order between them. Second, the relationship between BP level and NCDs, although explanatory, cannot be viewed as causal, because apart from the influence of BP level, NCDs also have to be influenced by many other risk factors.

The major strengths of our study are as follows: Firstly, we compare the previous and the latest BP definition and find higher prevalence of hypertension tends to cause more NCDs in middle-aged and elderly adults in China. This can raise more adults’ awareness to prevent high blood pressure and pay attention to regular and healthy lifestyle. This most complete picture of BP level and NCDs in middle-aged and older adults in china were not included in previous studies and are of clinical, public health, and health systems significance. Secondly, it is a nationwide survey in a developing country which is presently undergoing rapid urbanization and therefore provides a unique dataset with which to explore the effect of multiple demographic variables on the prevalence of hypertension and other NCDs. Furthermore, the survey instrument which was developed based on the best international practices, ensures the credibility of the results [17].
